# Thrips domiciles protect larvae from desiccation in an arid environment

**DOI:** 10.1093/beheco/aru128

**Published:** 2014-08-05

**Authors:** James D.J. Gilbert

**Affiliations:** ^a^Department of Evolution, Behaviour and Environment, John Maynard Smith Building, University of Sussex, Falmer, Brighton BN1 9QG, UK,; ^b^Department of Biology, University of Sydney, Sydney, NSW 2006, Australia, and; ^c^Fowlers Gap Arid Zone Research Station, School of Biological, Earth & Environmental Sciences, University of New South Wales, Sydney, NSW 2052, Australia

**Keywords:** cooperative breeding, humidity, moisture, nestbuilding, niche construction, parental investment, sociality, water balance.

## Abstract

Desiccation is a particular risk for small animals in arid environments. In response, many organisms “construct niches,” favorable microenvironments where they spend part or all of their life cycle. Some maintain such environments for their offspring via parental care. Insect eggs are often protected from desiccation by parentally derived gels, casings, or cocoons, but active parental protection of offspring from desiccation has never been demonstrated. Most free-living thrips (Thysanoptera) alleviate water loss via thigmotaxis (crevice seeking). In arid Australia, Acacia thrips (Phlaeothripidae) construct many kinds of niche. Some thrips induce galls; others, like *Dunatothrips aneurae*, live and breed within “domiciles” made from loosely glued phyllodes. The function of domiciles is unknown; like other constructed niches, they may 1) create favorable microenvironments, 2) facilitate feeding, 3) protect from enemies, or a combination. To test the first 2 alternatives experimentally, field-collected domiciles were destroyed or left intact. Seven-day survival of feeding and nonfeeding larval stages was monitored at high (70–80%) or low (8–10%, approximately ambient) humidity. Regardless of humidity, most individuals survived in intact domiciles, whereas for destroyed domiciles, survival depended on humidity, suggesting parents construct and maintain domiciles to prevent offspring desiccating. Feeding and nonfeeding larvae had similar survival patterns, suggesting the domicile’s role is not nutritional. Outside domiciles, survival at “high” humidity was intermediate, suggesting very high humidity requirements, or energetic costs of wandering outside domiciles. *D. aneurae* commonly cofound domiciles; cofoundresses may benefit both from shared nestbuilding costs, and from “deferred byproduct mutualism,” that is, backup parental care in case of mortality.

## Lay Summary

Tiny Acacia thrips build communal “domiciles” in arid Australia. Here I show that domiciles prevent desiccation, ensuring offspring survival—a novel kind of parental care in insects. We suspect many insect parents perform this function for offspring, but it has never been demonstrated experimentally. If larvae require parental care, this suggests one reason thrips are sometimes communal: if a female dies, her nestmates can ensure her offspring survive.

## INTRODUCTION

Desiccation is a common environmental hazard in terrestrial habitats (e.g., Schmidt-Nielsen 1997), most especially in arid zones (Whitford 2002). For insects and other small-bodied animals, with large surface area:volume ratios, desiccation risk in arid environments is acute (Schmidt-Nielsen 1984; Chown et al. 1995; Le Lagadec et al. 1998). Some carry physiological adaptations, such as desert beetles (Zachariassen 1996), but solutions can also be behavioral, such as fog or dew collection (Henschel and Seely 2008); and many create and maintain favorable microenvironments such as burrows (Henschel 1997), or plant galls (Crespi et al 2004), which render inhabitants relatively insensitive to changes in outside climate (Hadley 1970; Fernandes and Price 1992; Fay et al. 1993; Price et al. 1998; reviewed in Danks 2002)—a process known as niche construction (Odling-Smee et al. 1996).

Insect offspring are particularly sensitive to humidity, especially in arid zones, as they commonly lack a sclerotized cuticle (Kirk 1997). While most insects abandon offspring, many construct nests or otherwise actively modify the environment to help offspring develop (reviewed in Costa 2006). For diapausing eggs, parental arthropods sometimes produce or construct niches to reduce desiccation via, for example, cocoons in spiders (Hieber 1992 and references therein), the bags of bagworms (Rivers et al. 2002), oothecae in mantises (Birchard 1991), and a gelatinous matrix in Limnephilid caddisflies inhabiting vernal pools (Wiggins 1973). Although not traditionally viewed as “parental behaviour” per se (see Hinton 1981; Clutton-Brock 1991; Costa 2006), these parental traits would certainly fall under the expanded definition of parental care as “any parental trait that benefits offspring” provided by Smiseth et al. (2012, p. 7). Perhaps surprisingly, though, there has to my knowledge been no demonstration of active parental behavior protecting offspring from the effects of low humidity, although detailed observational data suggest that burrows dug by *Parastizopus* beetle parents (Coleoptera: Tenebrionidae) in the Kalahari play a role in preventing offspring drying out (Rasa 1995, 1998, 1999). Notably, digging to extend burrows into moist sand stops once the offspring’s cuticles have hardened (Rasa 1999).

One insect group whose members are especially prone to desiccation, owing to their tiny size, is the thrips (Thysanoptera) (Lewis 1962; Kirk 1997). The free-living majority of thrips worldwide are thigmotactic (seeking small spaces), which reduces desiccation risk (Kakei and Tsuchida 2000). Acacia thrips (Phlaeothripidae) inhabit the Australian Outback, and have evolved a range of solutions to problems posed by this semiarid to arid environment (Crespi et al. 2004). Members of one clade induce galls, or are kleptoparasites that attack and usurp gall inhabitants. Further species opportunistically inhabit abandoned galls (Crespi and Mound 1997). Members of a third radiation, quite separate from the gall-associated lineage, construct “domiciles.” Unlike galls, domiciles are not induced from plant tissue, but instead are built by gluing phyllodes (leaf-like projections of the stem) together loosely with silk-like anal secretions (henceforth “silk”) (Morris et al. 2002). As in the gall-associated lineage, domiciles are also attacked by related thrips lineages, by both kleptoparasites (Mound and Morris 1999) and inquilines (Gilbert et al. 2012).

**Figure 1:**
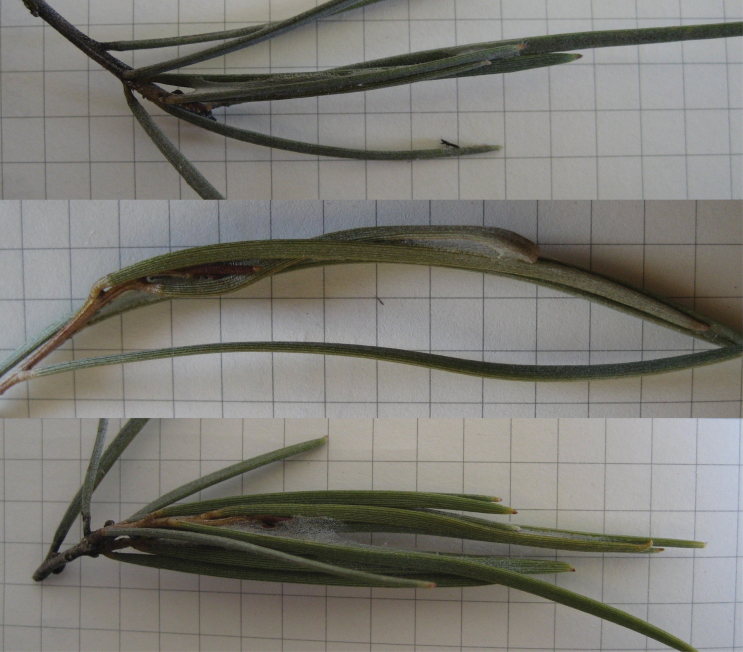
Examples of *Dunatothrips aneurae* domiciles on *Acacia aneura*. Squares measure 0.5×0.5cm.

Compared with their gall-inducing relatives, domicile-building thrips are poorly studied. The ecological function of the domicile is currently unknown. Morris et al. (2002, p. 472) suggested that, because the environment is arid, domiciles built by *Dunatothrips aneurae* Mound (Figure 1) are “critical for preventing desiccation,” a statement supported by circumstantial evidence. At ambient humidity, adults and larvae of all stages shrivel and invariably die when outside the domicile, usually within 48h, exhibiting symptoms of desiccation (inactivity, curling of legs, then shrivelling until paper-thin; Laughlin 1977) (Gilbert JDJ, personal observation). In contrast, inside intact domiciles in the field, individuals are recorded living more than 60 days (Gilbert JDJ, unpublished data), though the extent of their normal lifespan is currently unknown. It seems likely that, as with thigmotaxis, adaptations such as the domicile serve as protective buffers against the Outback’s inhospitable environment (Crespi et al. 2004), but this has not yet been formally tested.

In arthropods, parental care has diverse costs and benefits (e.g., Zink 2003; Gilbert et al. 2010, reviewed in Wong et al. 2013); parentally constructed niches (or nests) confer various benefits upon offspring (reviewed in Hinton 1981; Choe and Crespi 1997) which have traditionally been broadly classified into 3 nonmutually exclusive categories: protection against enemies, nutrition, and microenvironment (see e.g., Danks 2002; Stone and Schönrogge 2003). In this study, I conducted laboratory experiments with *D. aneurae* domiciles to test predictions arising from 2 of these: the “microenvironment” and “nutrition” hypotheses. Thrips are an excellent group for experimentally teasing apart nutrition-based explanations from alternative hypotheses, because the first 2 larval stages are feeding stages, whereas the final 3 are nonfeeding pupal instars, allowing us to dissociate the effects of nutrition from the effects of microenvironment. The biology of these different stages, and resulting expectations relevant to the hypotheses, are outlined in Table 1.

**Table 1:** Characteristics of larval and adult stages of Tubuliferan thrips

	Biology			Predicted sensitivity to reduction in:	
Stage	Feed?	Sclerotized?	Body size (SA:V ratio)	Food	Humidity
Early-stage larvae (instars I, II)	Yes	No	Small–medium (high)	Very high	Very high
Late-stage larvae (propupa, pupa I, II)	No	No	Large (low)	None	High
Adult	Yes	Yes	Large (low)	Moderate	High or moderate

In the experiment, field-collected domiciles were destroyed or left intact, and kept at high (70–80%, approximately optimal for many thrips; Kirk 1997) or low (8–10%, approximately ambient) humidity, and I monitored the survival of adults and larvae over 7 days. Specific predictions arising from “nutrition” and “microenvironment” hypotheses tested in this study are outlined in Table 2 (along with those from the “enemies” hypothesis for context). To test additionally whether adult presence is necessary for offspring survival, I repeated the experiment twice: once with and once without the adult present. If adults play an active role in offspring survival independently of domicile construction, such as food provisioning, then offspring should survive longer with adults present (Experiment 1) than with adults absent (Experiment 2). Results from both experiments supported the “microenvironment” hypothesis but not the “nutrition” hypothesis, providing the first experimental evidence of parents actively protecting offspring against low humidity.

**Table 2:** Experimental predictions arising from 3 common hypotheses of nest function

Hypothesis	Prediction^a^	Explanation
Enemies: domicile primarily protects against attack from enemies^b^	Main effect of humidity, no effect of treatment, (IH = DH) > (IL = DL)	Destruction of the nest should have no effect upon survival in a predator-free laboratory. Survival is expected to vary with humidity regardless of nest integrity
Nutrition: domicile primarily facilitates feeding by larvae and/or adults	Interaction between “treatment” and “developmental stage,” nonfeeding stages: (IH = DH) > (IL = DL)	Nonfeeding stages should be affected by humidity, but not by nest destruction as they do not need to feed
	Feeding stages: IH > DH and IL > DL; also:	Feeding stages in destroyed nests should die more quickly, as they are no longer confined to the nest (feeding) site by the nest wall
	IH > IL and DH > DL	Additionally, low humidity should reduce survival irrespective of nest integrity
Microenvironment: domicile primarily protects against low humidity	Effect of treatment only, (IH = IL) ≥ DH > DL	All individuals in intact nests should have high survival irrespective of humidity. After nest destruction, survival is expected to vary with humidity

Predictions with respect to survival within domiciles.

^a^Key: IH, intact, high humidity; IL, intact, low humidity; DH, destroyed, high humidity; DL, destroyed, low humidity.

^b^Note that the study was not designed to test the Enemies hypothesis, but its predictions are included for completeness.

## MATERIALS AND METHODS

In *D. aneurae*, females construct domiciles singly or in groups on *Acacia aneura* (Morris et al. 2002) throughout the eastern part of this plant’s range (Crespi et al. 2004), lose their wings (dealate), and live and breed entirely within the domicile. Males are probably present at founding, but do not help in nestbuilding and are expelled after mating (Gilbert and Simpson 2013), although they are sometimes seen inside immature domiciles (Gilbert JDJ, unpublished data). All feeding stages feed on the phyllode surface enclosed by the domicile and, as with most other thrips, offspring feed independently. Offspring develop into adults inside domiciles, most dispersing thereafter while some apparently remain and become dealate within the natal domicile (Bono and Crespi 2008); however, whether they then breed has yet to be established. Unlike other domicile formers such as *Paracholeothrips* (Crespi et al. 2004), there is no apparent defense by inhabitants against intruders in *D. aneurae* (Gilbert et al. 2012; Gilbert and Simpson 2013), and the domicile does not appear to be a site for (or otherwise facilitate) parental food provisioning—larvae appear to feed independently of adults, giving no indication of provisioning or social foraging as, for example, in *Anactinothrips* (Kiester and Strates 1984).

Both experiments were conducted during the austral spring (September to October) 2013. Experiment 1 focused on survival of larvae with adults present in intact versus destroyed domiciles. Thirty-two fully built, singly founded, mature *D. aneurae* domiciles containing a mix of larval stages were identified and collected from *A. aneura* trees in Bald Hills Paddock, Fowlers Gap, nr Broken Hill, NSW 2880, Australia (GPS: 30°57′40′′ S, 141°42′11′′ E). The *A. aneura* sprigs containing these domiciles, each approximately 5–15cm long, were gathered from the field. All experimental treatments were begun <6h after collection. Owing to xerophytic adaptations *A. aneura* dries out very slowly and sprigs were not appreciably wilted or dried by the end of the experiment. Domiciles were randomly assigned to treatments in a crossed design: In the first domicile treatment (I, “intact”), domicile silk was left intact and the adult and larvae were left in the domicile. The entire sprig containing the domicile was placed in a 50mL Falcon tube with the lid loosely screwed down to prevent thrips escaping while allowing humidity to equilibrate. The second domicile treatment was identical except that domicile silk was completely removed using watchmaker’s forceps (D, “destroyed”).

Each domicile treatment was replicated at 2 humidity treatments: high and low, by placing the tubes into incubators (Brinsea® Octagonal 40), one maintained at 70–80% r.h. (approximately optimal for most thrips development [Kirk 1997]; range during experiment 64.0–81.1%) and the other with its humidifier switched off and with parched rice added to reduce humidity to approximately 10% r.h. (range during experiment 8.1–10.1%, mirroring ambient humidity in the field which varied from 7% to 13%). Both incubators were maintained at approximately 26 °C, although on hot days both experienced the same degree of slight variation depending on ambient temperature (range during experiment 25–34 °C). For both domicile treatments, half the tubes were randomly assigned to the “high humidity” treatment (H), while the other half were assigned to the “low humidity” treatment (L), thus giving 4 treatment groups: IH1, IL1, DH1, and DL1.

Experiment 2 focused on larval survival without adults. Sixteen mature, singly founded domiciles containing a mix of larval stages were gathered from the same location as for experiment 1. The adult was removed from each domicile along with any mature adult offspring present, since these have been observed assisting with domicile repair (Gilbert JDJ, unpublished data) and may also contribute to larval survival in other unknown ways. Adults were removed using a coarse hair probe by carefully peeling back a small portion of the nest wall and replacing it afterward. In the first domicile treatment, larvae were left in their intact domicile of origin (I, “intact”). In the second domicile treatment (D, “destroyed”), 1–5 larvae were extracted from the intact domicile of each I group using a coarse hair probe and placed in a separate tube, creating a paired design. To ensure that larvae in the D treatment (like those in the I treatment) also had access to a viable feeding site (i.e., a site chosen for a successful domicile), extracted larvae were placed directly onto the site of a different domicile collected alive from the field, whose adult, larvae and silk had been completely removed. As in experiment 1, for both domicile treatments, half the tubes were randomly assigned to the high humidity treatment (H), while the other half were assigned to the low humidity treatment (L), thus giving 4 treatment groups: IH2, IL2, DH2, and DL2. Note that, while it was attempted to randomize larvae among treatments, I discovered that larvae introduced to foreign intact domiciles tended to wander out of them, making this design impractical. Thus, experiment 2 is unbalanced from the point of view of disturbing the larvae, and I acknowledge that this could be a source of experimental bias.

To perform regular observations, I removed tubes temporarily from the humidifiers (<60 s) and examined the tube and sprig under a binocular microscope (Nikon® SMZ745T). Intact domiciles were examined by carefully peeling back a small portion of the silk using forceps (the same portion used to remove individuals, where applicable), then replacing it after observation. A powerful LED torch (LED Lenser®), shone from behind the domicile, was also used to observe activity through the silk in inaccessible parts of the domicile. I checked all domiciles at 1, 2, 4, and 6h, after which I checked them every 6h, recording 1) the survival of adults and larvae in experiment 1 and larvae in experiment 2; 2) whether the adults or larvae in the I groups had left the domicile in both experiments; 3) whether individuals in the D groups had remained at the site of their original domicile in both experiments; and 4) any larvae moulting into adults. The experiments were terminated at 182h (7.5 days; experiment 1) and 120h (5 days; experiment 2).

Survival of adults and larvae were modeled using Cox mixed-effects models using the coxme and survival packages (Therneau 2012, 2013) in R 3.0.1 (R Core Team 2013). In experiment 1 I included “treatment” (IL, IH, DL, or DH), “developmental stage” (feeding, nonfeeding, adult) and their interaction as fixed terms and “domicile ID” as a random term. In experiment 2, which had a paired design, I additionally included “tube” as a random term nested within “domicile ID.” I checked the proportional hazards assumption for all models by visual inspection of the hazard function and by using the cox.zph() function in R. I used a reverse stepwise approach to select the best model, comparing nested models against each other with likelihood ratio tests (assumed to have a chi-squared distribution). Larvae sometimes exited the domicile in the I groups; these were excluded from analyses and their survival outside the domicile was modeled separately. A few larvae drowned in condensation droplets in the high humidity treatments; data for these were censored at the point of drowning.

For the “developmental stage” term in each model, I analyzed nonfeeding larval stages (pupal instars) separately from feeding stages (nymphal instars) and from adults. An exclusively “nutritional” hypothesis (Table 2) predicts an interaction between “treatment” and “larval stage” with nonfeeding stages experiencing little or no effect of nest destruction, but feeding stages suffering high mortality outside of destroyed nests; and that individuals inside intact nests will suffer higher mortality at low humidity than at high humidity. By contrast, an exclusively “microenvironment” hypothesis predicts that individuals inside intact nests will suffer no consequences of low humidity, and that feeding and nonfeeding stages will experience similarly deleterious effects of nest destruction (i.e., no interaction of “treatment” and “larval stage”), with the DL group having uniformly high mortality. To test these predictions, I used the minimal model of survival to assess the interaction of “treatment” with “larval stage” and also performed 2 planned orthogonal contrasts: first, IH against IL (predicted to be similar under the microenvironment hypothesis, but IH > IL under the nutrition hypothesis), and second, DL against pooled data for DH, IH, and IL (DL < [DH, DL, IL] for all larval stages for the microenvironment hypothesis, but only for feeding stages in the nutrition hypothesis) (Table 2).

## RESULTS

### Experiment 1 (larval survival with adults present)

In the experiment focusing on both adults and larvae together, stepwise term deletion revealed that the minimal model contained “treatment” alone. There was no significant “treatment” × “stage” interaction (dropping this term, *χ* ^2^ = 5.71, Δdf = 6, *P* = 0.45) nor an effect of dropping “stage” (*χ* ^2^ = 4.40, Δdf = 2, *P* = 0.12), but dropping “treatment” significantly reduced the explanatory power of the model (*χ* ^2^ = 65.5, Δdf = 3, *P* < 0.0001; Table 3, experiment 1). Thus, adults, feeding, and nonfeeding larvae did not statistically differ in their response to treatments, but treatments differed from each other.

**Table 3:** Model tables for minimal models in experiment 1 and experiment 2

	Coefficients			Model testing		
(a) Experiment 1	β	SE (β)	Exp (β)^a^	*χ* ^2^ (LRT)	df	*P*
Fixed term						
Treatment				65.51	3	<0.0001
IH1	0	—	1			
IL1	1.580	1.096	4.853			
DH1	4.186	1.035	65.746			
DL1	5.544	1.056	255.747			
Random term	SD	Variance				
Domicile	0.0091	0.0001		0.004	1	0.94
(b) Experiment 2						
Fixed term						
Treatment				48.53	3	<0.0001
IH2	0	—	1			
IL2	^b^	^b^	^b^			
DH2	2.423	0.774	11.28			
DL2	3.719	0.822	41.23			
Random term	SD	Variance				
Domicile	0.2695	0.0727		<0.001	1	0.98
Tube within domicile	0.0197	0.0004		0.11	1	0.73

For details of planned orthogonal contrasts, see text. Fitting all models again without the random term (using the coxph function in the survival package in R; Therneau 2013) had no appreciable effect upon the explanatory power of each respective model, indicating that variance in survival did not differ among domiciles; the minimal model was the same in both cases. SD, standard deviation; SE, standard error.

^a^Equivalent to the hazard ratio compared with the baseline treatment level (in this case IH). In this case “hazard” pertains to mortality, higher values of β indicating higher risk of death.

^b^Inestimable as all individuals survived, that is, were censored.

**Figure 2:**
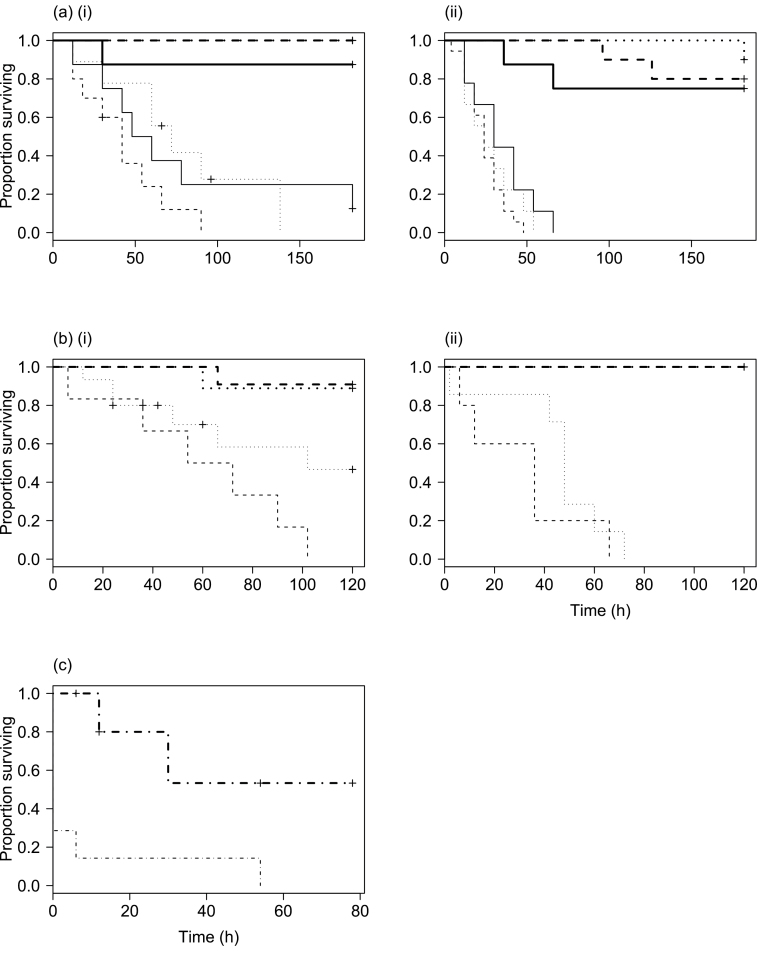
Survival curves for experimental treatments. (a) Experiment 1: (i) high humidity, (ii) low humidity; (b) Experiment 2: (i) high humidity, (ii) low humidity; (c) All larvae exiting intact domiciles (pooled across experiments; *X* axis shows time since emergence from domicile). Key: Thick line, intact domicile treatment; thin line, destroyed domicile treatment; solid line, adults; dashed line, feeding larval stages (larvae I and II); dotted line, nonfeeding larval stages (propupa, pupa I, pupa II); dot-dashed line, all larvae combined. Crosses show censored data points. (Nb. in (a)(i) and (b)(ii), dashed and dotted lines for intact domicile treatments are superposed, as all larvae survived).

Almost all individuals remaining inside intact domiciles (IH1 and IL1) survived until experiment 1 was terminated at 182h, regardless of humidity (Figure 2a). In contrast, all individuals in the DL1 group had died by 66h (median time to death 24h for feeding larvae, 24h for nonfeeding larvae, 30h for adults). Individuals in the DH1 group showed intermediate survival (median time to death 42h for feeding larvae, 72h for nonfeeding larvae, 54h for adults) and had all died by 182h.

In the planned treatment contrasts, contrast 1 (asking whether survival in intact domiciles was sensitive to humidity, comparing the intact groups, IH1 vs. IL1) was not significant (*z* = −1.44, *P* = 0.15), whereas a significant result was obtained for contrast 2 (asking whether exposed thrips die more quickly at low humidity, comparing DL1 versus pooled data for others, *z* = −7.63, *P* < 0.0001).

### Experiment 2 (larval survival without adults present)

In the experiment focusing on larvae without adults, the minimal model again contained treatment alone. There were no significant effects of dropping the “treatment” × “stage” interaction (*χ* ^2^ = 0.62, Δdf = 3, *P* = 0.89) nor of dropping “stage” (*χ* ^2^ = 2.23, Δdf = 1, *P* = 0.14) but again dropping “treatment” had a highly significant effect upon model fit (*χ* ^2^ = 48.52, Δdf = 3, *P* < 0.0001; Table 3, experiment 2). Again, almost all individuals remaining inside intact domiciles (IH2 and IL2) survived until the experiment was terminated at 120h, regardless of humidity treatment (Figure 2b), while the DL2 group had all died by 72h (median survival 36h for feeding larvae, 48h for nonfeeding larvae). In the DH2 group, survival was again intermediate (median survival 54h for feeding larvae, 102h for nonfeeding larvae).

In the planned treatment contrasts, contrast 1 (IH2 vs. IL2) was formally inestimable because, in the IL2 group, all data points were censored, that is, none of the 12 larvae remaining inside the domicile died. However, I can reasonably infer from this fact, and that only 2/20 larvae died in the IH2 group, that the 2 groups were not different. On the other hand, a highly significant result was seen for contrast 2 (DL2 vs. pooled data for others, *z* = −5.26, *P* < 0.0001).

### Larvae exiting intact domiciles in the intact groups

In the “Intact” groups of both experiments, any larvae that exited intact domiciles died rapidly (median survival 30h, pooled across experiments and treatments), but almost all larvae that chose to remain inside intact domiciles survived the entire experiment (only 3 out of 38 larvae died inside intact domiciles in experiment 1; 2 out of 32 in experiment 2). Larvae exited intact domiciles relatively infrequently. More larvae exited intact domiciles after adults had been removed (number exiting domicile, pooling across treatments, experiment 1, 5 larvae; experiment 2, 15 larvae; *χ* ^2^ = 5, Δdf = 1, *P* = 0.02). As expected from the findings presented above, larvae died more quickly after exiting domiciles in the low humidity treatment than in the high humidity treatment in both experiments (pooled across experiments, median survival in L groups 0h [i.e., were typically dead upon discovery outside the domicile]; in H groups 54h; Cox proportional hazard model with no random effect: experiment 1, *χ* ^2^ = 4.60, Δdf = 1, *P* < 0.05; experiment 2, *χ* ^2^ = 8.56, Δdf = 1, *P* < 0.01; combined data, *χ* ^2^ = 9.83, Δdf = 1, *P* < 0.01, Figure 2c).

### Behavior and adult/offspring interactions

In the DH and DL groups in both experiments, neither adults nor larvae remained at the site of the destroyed domicile but instead wandered apparently aimlessly over the plant sprig and the wall of the tube. Adults in DH1 and DL1 groups frequently returned to the site of the destroyed domicile and sometimes attempted to rebuild the domicile, although none laid down more than a few strands of silk and were often seen sheltering in small spaces between phyllodes or in the crook of the phyllode petiole. Larvae in DL and DH groups were never seen sheltering in this way, never returned to the destroyed domicile site, and, as in prior observations (e.g., Gilbert & Simpson 2013), were never seen to produce any silk. Adults were never seen engaging in any obvious interactions with offspring, whether outside or inside the domicile. In the IL1 and IH1 groups, larvae exiting intact domiciles were not guided back by adults. Adults in these groups partially repaired the minor damage to the domicile caused by my peeling back, and subsequent replacement of, part of the silk wall to allow temporary observation (the dynamics of repair behavior will be addressed in a future manuscript).

### Larvae becoming adult

I did not observe enough larvae becoming adults for statistical comparison among groups; hence I cannot form conclusions about the effect of humidity upon long-term viability in *D. aneurae*. However, 3 individuals from intact domiciles became teneral adults in each of experiment 1 and experiment 2 and were all still alive at the end of their respective experiments; there is no reason to suppose they were not viable individuals. In destroyed domiciles, 4 individuals (2 from each experiment) became teneral, but all died shortly thereafter.

### Support for hypotheses

The “nutrition” hypothesis (Table 2) predicted that nonfeeding and feeding larval stages would differ in their response to the treatments: feeding larvae would die from starvation after wandering outside a destroyed domicile, whereas nonfeeding larvae would not be affected by nest destruction, their survival varying only with humidity. This was not supported, as nonfeeding larval stages were just as susceptible to nest destruction as feeding stages, and were similarly insensitive to low humidity inside intact domiciles (Figure 2, Table 3).

The “microenvironment” hypothesis predicted that individuals of all stages whose nests were destroyed would survive longer at high humidity than at low humidity, whereas inside intact nests, survival would be similarly high regardless of humidity. This prediction was supported (Figure 2, Table 3).

## DISCUSSION

A large proportion of arid zone invertebrates construct microhabitats in the form of domiciles, galls, or burrows (e.g., Price et al. 1998). *D. aneurae* were sensitive to humidity while wandering outside of a domicile (whether experimentally or having chosen to exit the domicile), surviving longer at higher humidity. By contrast, inside intact domiciles, individuals of all stages had high survival regardless of humidity outside. Because adults are the only stage that produces silk, construction and maintenance of the domicile by adults is clearly necessary for larval survival and can be considered “parental care.” The observed patterns did not change in the absence of adults, demonstrating that adult presence is not strictly required in the short term for larval survival. However, domiciles are very frequently damaged by wind in the field, often with loss of all inhabitants (Gilbert JDJ, unpublished data), but are usually repaired by adult residents, implying that not only domicile building but also domicile maintenance is necessary for larval survival. Parental care is therefore likely to be progressive, that is, requiring female presence throughout larval development.

Nutrition is an alternative candidate function for the *Dunatothrips* domicile (Table 2). For example, preferred oviposition sites may represent areas of particularly high nutritional value (Sadeghi and Gilbert 1999; Pöykkö 2006; Refsnider and Janzen 2010) where it is beneficial to confine larvae, or which are specially prepared by parents for larvae (e.g., Eggert et al. 1998). However, survival followed similar patterns for both feeding and nonfeeding stages of larvae within the same domicile. This suggests that mortality was not due to differences in the nutritional environment inside and outside of a domicile, which would have differentially affected the feeding stages of the life cycle (early larvae and adults).

Defense against enemies may also be a candidate function for domiciles (Table 2). It was not the goal of the present study to address whether or not domiciles provide any degree of protection at all from enemies—rigorous testing would involve assessing the success of experimentally induced attacks. However, survival outside domiciles was reduced even in the predator-free conditions of the lab, and I can thus reject an exclusively protective function for domiciles. Recent evidence shows that domiciles present no particular barrier to entry by prospecting conspecific males (Gilbert and Simpson 2013). Known enemies of *D. aneurae* are all thrips of similar size to the hosts, which presumably would also have little problem entering domiciles—including kleptoparasites (Mound and Morris 1999) and inquilines, the latter of which are generally unchallenged by domicile inhabitants (Gilbert et al. 2012). In the field, adults have never been observed defending against intruders (Gilbert and Simpson 2013), although such behavior has been observed in domicile-building *Paracholeothrips* (Crespi et al. 2004).

In this experiment, all individuals in destroyed nests were seen to wander around their experimental cage, activity that may have carried unquantified energetic costs. I could not formally distinguish between the effect of destroying the nest and the energetic costs of such wandering, but I note that individuals wandering outside nests did not incur a uniform survival cost. Rather, individuals in the “destroyed, low humidity” treatment died sooner than those in the “destroyed, high humidity” treatment, so survival was at least affected or mediated by humidity, whereas this was not the case inside intact nests. Nevertheless, activity accelerates water loss at low humidity (Willmer 1982), and minimizing unnecessary activity by larvae may be one way that domiciles reduce desiccation—although this is highly unlikely to be the domicile’s main function. The costs of wandering around the tube after domicile destruction may also explain why, even at high humidity, survival of individuals outside destroyed domiciles was lower than that inside intact domiciles. An alternative explanation may be that 70–80% humidity may be lower than optimal for development in *D. aneurae*, despite being optimal for other studied thrips species. Higher humidities are generally required at higher temperatures (Lewis 1962; Laughlin 1977; Kirk 1997); the heat of the Australian arid zone may create especially high humidity requirements.

Thus, the results I present here support the assertion by Morris et al. (2002) that protecting against low humidity may be an important benefit of *Dunatothrips* domiciles in the Outback. However, the precise mechanism(s) by which domiciles combat low humidity are still a focus for research. One likely possibility is that domiciles may elevate local humidity, perhaps by trapping moisture from transpiration and/or respiration by the plant, which could be simply tested using a microhygrometer. Additionally, domiciles may restrict the movement of inhabitants, reducing activity costs that are higher at low humidity. Dissociating these 2 effects requires further research.

Alternative methods of niche construction, such as insect galls, are also thought to play a role in combatting low humidity in arid zones, which may explain the preponderance of galls in these ecosystems (Price et al. 1998; Carneiro et al. 2005; Bairstow et al. 2010). It is likely that protection from desiccation is also just as important a benefit in species with nests, hives, and burrows (e.g., Human et al. 2006), especially in arid zones (Rasa 1990). The role of humidity in facilitating invasion of arid zones by nestbuilding or burrowing species is therefore a question for further research. Larvae of many thrips in temperate climates pupate at ground level where humidity is higher than on host plants (Kakei and Tsuchida 2000; Steiner et al. 2011) and there is evidence that ambient humidity affects the decision whether to pupate on the ground or on the plant (Steiner et al. 2011). Long-term studies of soil moisture and *Thrips imaginis* development in Australia in the 1930s initially suggested that pupation at ground level might not be an option for thrips in arid zones (Mound 2013). Although this picture may well be simplistic (Mound 2013 and personal communication), it is reasonable to hypothesize that lack of soil moisture may necessitate alternative strategies for larval development in arid zones. Domiciles and galls may thus represent alternative solutions to this problem by separate lineages of thrips invading the arid Outback. It remains to be investigated how kleptoparasites, for example, *Xaniothrips* (Mound and Morris 1999; Bono 2007) survive within usurped domiciles, having expelled or killed the hosts, and with currently no evidence suggesting they can produce silk to repair damage.

### Presence of adults

Almost all larvae that chose to remain inside intact domiciles survived irrespective of adult presence. In both field and laboratory, though, adults will actively repair damaged domiciles (Gilbert and Simpson 2013 and unpublished data), which may reduce desiccation and keep larvae from falling onto the ground to an inevitable death. However, even in the benign conditions of the lab, with no chance of damage to intact domiciles, more larvae chose to exit the domicile when adults were absent (i.e., in experiment 2) than with adults present. There was no guiding or restraining behavior by adults when larvae exited domiciles (either here or in extensive prior observations; Gilbert JDJ, personal observation). Speculatively, adults may emit volatile aggregation pheromones within domiciles (Wertheim et al. 2005). Various Thripidae exhibit sex pheromones (Hamilton et al. 2005; Webster et al. 2006) and alarm pheromones may occur in gall-inducing Phlaeothripinae (De Facci et al. 2013) but to my knowledge no aggregation pheromones are known in thrips, so this possibility may warrant further attention.

### Implications for social evolution

*D. aneurae* are also known to exhibit facultative pleometrosis (joint nestbuilding; Morris et al. 2002). Bono and Crespi (2006) demonstrated survival benefits of this behavior: cofoundresses are more often found alive than singletons, especially following attack by kleptoparasites. In the light of the findings I present here, speculatively, 2 further benefits of joint nesting are possible which would be loosely analogous to the “fortress defence” versus “life insurance” framework proposed for eusocial insects by Queller and Strassmann (1998) and together contain elements of both kinds of social benefit. First, for a given domicile size, cofoundresses may spread the cost of domicile construction and maintenance, thus obtaining a valuable protective resource (a domicile) for less individual cost. Second, cohabiting foundresses may benefit from “deferred byproduct mutualism” (Kukuk et al. 1998). If a cofounding female dies with offspring still undeveloped, other females can perform parental care in her place, securing her initial investment. Noneusocial, communally breeding bees are thought to gain such benefits, stabilizing otherwise risky foraging behavior (Kukuk et al. 1998). In the same way, *D. aneurae* larvae need adults present to maintain domicile integrity or they will dry out; several females may provide mutual backup for each other’s offspring. If this latter benefit were to operate, it would require that domicile maintenance is costly or risky to adult thrips; this has not been demonstrated here and the possibility requires further research. However, the requirement for parental care in *D. aneurae* adds to the parallels that have already been pointed out between domicile-building thrips and the primitively eusocial Hymenoptera (reviewed in Bono and Crespi 2008). Progressive parental care is thought to have been crucial to the evolution of social behavior in Hymenoptera (e.g., Field and Brace 2004); elucidating the evolutionary associations between parental care and pleometrosis in *Dunatothrips* may provide key insights into the evolution of joint nesting in this genus.

Housekeeping roles such as nest maintenance are typical of individuals that are reproductively subordinate within cooperative groups (Benton and Foster 1992; Kurosu et al. 2003; Biedermann and Taborsky 2011). In thrips, domicile construction and maintenance via silk production is necessary for survival in their low humidity environment, but is also likely to carry costs to those performing this task. Thus, we might reasonably hypothesize that the tasks of reproduction and nest construction/maintenance might not be shared equally, an idea that now requires testing experimentally. Elucidating the costs and benefits of the relative contributions of thrips cofoundresses to reproduction versus domicile maintenance, along with cofoundresses’ genetic relatedness, will shed considerable light upon evolutionary routes to communal breeding in this species. *D. aneurae* cofoundresses comprise a mixture of related and unrelated individuals (Bono and Crespi 2008), reproductives and nonreproductives, and individuals that contribute and do not contribute to domicile maintenance (Gilbert JDJ, unpublished data) which will provide a rich testing ground for future research.

## FUNDING

This study was funded by the European Community’s Seventh Framework Programme (FP7/2007–2013) under grant agreement no: PIOF-GA-2011–299506.
